# Being Perceived and Being “Seen”: Interpersonal Affordances, Agency, and Selfhood

**DOI:** 10.3389/fpsyg.2020.01750

**Published:** 2020-07-30

**Authors:** Nick Brancazio

**Affiliations:** School of Humanities and Social Inquiry, Faculty of Arts, Social Sciences, and Humanities, University of Wollongong, Wollongong, NSW, Australia

**Keywords:** social affordances, interpersonal affordances, direct perception, social cognition, agency, selfhood

## Abstract

Are interpersonal affordances a distinct type of affordance, and if so, what is it that differentiates them from other kinds of affordances? In this paper, I show that a hard distinction between interpersonal affordances and other affordances is warranted and ethically important. The enactivist theory of participatory sense-making demonstrates that there is a difference in coupling between agent-environment and agent-agent interactions, and these differences in coupling provide a basis for distinguishing between the perception of environmental and interpersonal affordances. Building further on this foundation for understanding interpersonal affordances, I argue that in line with some enactivist work on social cognition, interpersonal affordances ought to be considered as those that are afforded by agents and are recognized as such. Given this distinction, I also make the point that because our social conventions establish persons as more than mere agents, the direct perception of interpersonal affordances may also involve seeing others as embodied selves. Distinguishing between types of affordances thus also matters ethically: there can be harms done when an agent is not perceived as an agent, and there can be harms done when an agent is not perceived as a self.

## Introduction

Are ecological psychology and enactivism committed to a difference between our perception of the environment and our perception of other agents? Drawing from James Gibson’s work (J. Gibson, 1979/2015) on perception, contemporary enactivism and ecological psychology both use the theory of affordances or perceived possibilities for interaction. Affordances are neither properties of the environment nor the agent, but are co-constituted in the agent-environment relationship, given the agent’s values, abilities (Chemero, 2003), and skills (van Dijk and Rietveld, 2017) as the agent actively explores her world (J. Gibson, 1979/2015). Ecological psychology is largely built around the notion of affordances as the main objects of perception, while in enactivism affordances have played a more subsidiary and contentious role.

Increasingly, enactivists are using the language of affordances in their explanatory frameworks (see, e.g., Gallagher, 2008, 2017; Di Paolo et al., 2017). Enactivism and ecological psychology share a number of theoretical commitments, and many see them as kindred approaches to cognition. Both reject the received view of cognition as internal, computational, and representational. Both propose that we see cognition as an active process constituted in the relationship between organism and environment. Both argue that perception is intersubjectively developed (Gallagher, 2008; De Jaegher et al., 2016), learned (E. Gibson, 1963), and/or socially mediated (Heft, 2007). These should be thought of as broad agreements in spirit, though, rather than precise overlaps – the approaches are sisters, not twins.

Given that the ecological approach relies on James Gibson’s theory of direct perception (J. Gibson, 1972/2002), we should understand affordances not as inferred through our perception of the environment, but as directly perceived. We see an apple *as* edible, rather than post-perceptually *inferring* that it is edible (for example, Nanay, 2011). Further, while apples can offer the possibility of sustenance or *afford* being eaten, this might only be perceived as a relevant affordance if an agent is actively searching for something to eat; if I were looking for something to hold down a paper that was in danger of blowing away, an apple might instead afford the possibility of serving that purpose for me.

The social contributions to affordance perception have been widely discussed and debated in the ecological psychology literature (e.g., Reed, 1991; Costall, 1995, 2012; Heft, 2007). Other people, though, are not apples, and how we perceive the affordances offered by other agents is a much smaller subset of this literature. The contemporary hybrid theory of ecological-enactivism has offered some headway on how we might approach uniquely *social* affordances (Rietveld, 2008; Rietveld et al., 2017), holding that social affordances offer possibilities for social interaction. However, ecological-enactivists have also maintained that there is an equivalence between our perception of environmental affordances and social affordances (Rietveld et al., 2013, 2017). This work on social affordances has been valuable for explaining how we might both pre-reflectively experience and conscientiously shape our interactive spaces.

Here, though, I propose that in bringing together ecological and enactivist views on social interactions, we need to maintain a finer-grained distinction between environmental affordances that offer opportunities for socializing, such as public spaces, and those offered by agents themselves. That is, I will argue that the perception of interpersonal affordances (Trierweiler and Donovan, 1994; Richardson et al., 2007; Fiebich, 2014), defined as opportunities afforded by other agents, is indeed different from the perception of environmental affordances, given what enactivism has provided on the unique nature of agent-agent coupling.

Given the role that intersubjectivity plays in the enactive framework, and the importance of joint sense-making in interaction (De Jaegher, 2013a,b), distinguishing between affordances in agent-environment and agent-agent relationships ought to be taken as both explanatorily and ethically relevant due to the differences in cognitive activities and types of coupling. Put simply, the perception of interpersonal affordances is uniquely interactive. While this is a foundational point for enactivist accounts of social cognition (Gallagher, 2008; De Jaegher, 2009), I argue here that this ought to be equally applicable when accounting for the perception of affordances offered by other agents.

Importantly, this distinction is also ethically relevant. For human forms of life, the mutual attribution of agency that happens in social interactions involves many layers. One of these, I argue, is that we perceive other humans as selves. Selves are scaffolded by social convention and practice and are developed in relation with others (Kyselo, 2014). Here, using Maiese’s “life-shaping” thesis of selfhood (Maiese, 2019), I show the importance of perceiving both agency and selfhood in interactions and, conversely, demonstrate the harm that can be done by refusing to recognize another as an agent or as a self. This advances the discussion on the ethical dimensions of affordances in interaction and helps illustrate the damage that is done when one is perceived as affording possibilities for interaction that deny their agency or aspects of their selfhood.

## Social Affordances and Interpersonal Affordances

The social aspects of affordances have been detailed in ecological psychology by those such as Heft (2007), who argues that the perception of affordances is in all ways social. That is, Heft argues that both the ontogeny and phylogeny of how we come to perceive affordances, for humans, is socially developed through niche construction and the influence of culture through the constructed ecological niche (see also: McGann, 2014 on intersubjectivity, E. Gibson, 1963 on perceptual learning, and Ramstead et al., 2016 on cultural affordances)^1^. The intersubjective development of affordance perception applies to both environmental affordances and the account of interpersonal affordances that I will offer here.

As Rietveld et al. (2017, p. 300) define them, social affordances are “possibilities for social interaction or sociability provided by the environment.” They have been defined elsewhere even more broadly:

“Social and communicative affordances that reflect the meaning of human activity for other humans (cf. McArthur and Baron, 1983; Reed, 1988). These include not only the affordances of symbolic behavior such as human conversation and writing (Dent, in press) but also the affordances of nonsymbolic activity such as facial expressions (Alley, 1988; Buck, 1988), gesture (Tomasello, 1988; Van Acker and Valenti, 1989), body postures and movements (Runeson and Frykholm, 1983), tone of voice (Walker, 1982; Walker-Andrews, 1986), and the direction of gaze (orienting; Scaife and Bruner, 1975; Butterworth and Cochran, 1980) that provide information about the actor as well as about other aspects of the environment. The symbolic behaviors (language) are entirely conventional and culture-specific, whereas the nonsymbolic are only partly so” (Loveland, 1991, p. 101).

Loveland’s conception incorporates a list of affordances that might be related to acts of socializing or communication. Loveland’s list is meant to be more limited than, for example, saying that affordances can be canonical, a term used by Costall (2012) to refer to the way that affordances can be specific to socio-cultural practices. That is, Costall uses this term to point out that some affordances are available only because those perceiving them have learned certain ways of engaging with the environment or certain meanings of items through social means. An example of this is a recycling bin. This only affords the recycling of an item if one has been raised in a social environment where recycling is a norm or somehow otherwise knows about the social convention of recycling.

Gallagher and Ransom (2016) use the term “social affordances” in an even more limited sense in discussing the social affordances provided by social media. As many of our social interactions do not take place in person, that a certain website or app affords sociability could mean many things. For example, an app can be used for facilitating meet-ups in the sense of one creating or responding to a social media event for an upcoming gathering or collective action. It could also mean facilitating direct exchange between agents in a virtual space, such as with a messaging app. This usage of the term is also becoming widespread in areas that study human-technology interaction and mediation, such as networking technology (e.g., Bradner, 2001) and social robotics (e.g., Paauwe et al., 2015).

Social affordances have also been discussed in some detail by ecological-enactivists. The hybrid theory of ecological-enactivism (Rietveld and Kiverstein, 2014) has brought together both the ecological and enactive approaches in their proposal of the Skilled Intentionality Framework (SIF; van Dijk and Rietveld, 2017). The SIF incorporates the “lived perspective of a skilled individual” as integral for understanding how it is that we perceive *relevant* affordances (van Dijk and Rietveld, 2017, p. 3). The development of the skills for being attuned to relevant affordances for the agent can be thought of as “multiple bodily states of action readinesss reciprocally coupled to the landscape of affordances, in the sense that these states of action readiness self-organize and shape the selective openness to the landscape of affordances” (van Dijk and Rietveld, 2017, p. 8). Though we might think of skill in the sense of expertise, this includes any embodied or pre-reflexive skills or capacities for navigating the world. Skilled intentionality can be as simple as selectively perceiving a mug handle as graspable when one is heading to the coffee pot for a refill. Through our skills and habits of coupling, we are selectively open to the relevant affordances of the environment for the task(s) we are undertaking.

In their discussion of social affordances, Rietveld et al. (2017) offer a number of concrete suggestions for improving sociability in the sense of providing spaces where people from disparate backgrounds or with very different interests might be inclined to come together. Their suggestions include park planning and other architectural interventions to offer options for activities conducive to social interactions in public spaces. In this sense, sociability could also be afforded anywhere that people tend to have social interactions, such as coffee shops, parks, the grocery store check-out lane, or even the sidewalk, though all of this would be heavily dependent on sociocultural norms and practices.

Because sociability and social interactions are quite different, it is important to distinguish these further. Affording sociability might apply to an area or an artifact (such as an app), in that it can lead to a social interaction, but these affordances themselves are not socially interactive in an interpersonal sense. A sociability affordance might be one that affords an interaction conducive space or can facilitate or lead to an interaction. These might often be a pre-cursor to a social interaction but are neither necessary nor sufficient to lead to an interaction. More specific claims about what is afforded by certain types of social affordances, or when sociability is afforded, should be made cautiously though. What one feels is an “interaction conducive space” would of course be dependent on culture, social position, and identity. There may be gender, race, neurodiversity, disability-related, or historical issues or dynamics that would influence whether spaces are perceived as hostile, dangerous, or uncomfortable for some and welcoming or comfortable for others (De Jaegher, 2013a,b; Heras-Escribano, 2019, Ch. 7; Jurgens, 2020).

It should also be stressed that, in the terms of the social cognition literature, interpersonal affordances should not be taken to imply a Theory of Mind, which is an inference about or simulation of the mental state of the other. A Theory of Mind is built on the idea that we are at a remove from the mental state of the other in social interactions, and that we use simulation (implicit mental simulation, e.g., Goldman and Sripada, 2005, or mirror neuron systems, e.g., Gallese, 2005) or inference (e.g., some kind of implicit or explicit theory about others’ minds, e.g., Gopnik and Wellman, 1992) to explain how we as spectators (Schilbach et al., 2013) come to know the other’s mental state (their intentions, emotions, etc.). Rather, perceiving an interpersonal affordance should be thought of as phenomenologically immediate, as with James Gibson’s theory of the direct perception of affordances (J. Gibson, 1979/2015).

Direct perception is the basis of interaction theory, the theory of social cognition proposed by Gallagher (2008). It might be helpful to draw a similarity between Gallagher’s direct perception theory and how we ought to understand interpersonal affordances. This enactivist conception of direct perception is built on the idea that cognition is fundamentally embodied and action-oriented. As such, it is not the case that mental states are locked away inside the mind of the other. In direct perception, we simply see affective states and goal-oriented actions as such, with no need for inference. Having moved away from the input-model of perception, there is no need for an inference or for simulation in order to see a motion of a hand toward a cup *as reaching for the cup*. Likewise, we see a friend *as* excited without need for inference or attribution (Varga, 2018). Reflexively, we might *make* this attribution, but in most cases this is because that is *how we perceived the action*. And while we might sometimes use an inferential process to try to figure out what someone is doing or feeling, this is when something is complex or confusing. It is the exception, not the rule.

Interaction theory incorporates affordance perception into the explanation of how it is that we directly perceive these mental states. This is explained by Gallagher and Varga (2014, p. 189):

“According to [interaction theory] and the direct perception hypothesis, social perception is enactive. That is, my perception of your action is already formed in terms of how I might respond to your action. I see your action, not as a fact that needs to be interpreted in terms of your mental states, but as a situated opportunity or affordance for my own action in response. The intentions that I can see in your movements appear to me as logically or semantically continuous with my own, or discontinuous, in support or in opposition to my task, as encouraging or discouraging, as having potential for (further) interaction or as something I want to turn and walk away from.”

While this is an excellent example of a fruitful integration of affordances with enactive approaches to social perception, interaction theory has been criticized for not being interactive enough (De Jaegher, 2009). In the following section, I will turn to the theory of participatory sense-making (De Jaegher and Di Paolo, 2007) to provide a more detailed argument, based in interaction, for holding that the perception of interpersonal affordances is different from the perception of environmental (and social or sociability) affordances due to differences in coupling.

## Enactive Autonomy and Interaction

Interpersonal affordances offer opportunities for interaction with another agent and, therefore differ in definition from environmental or sociability affordances. The participatory sense-making framework, grounded in autopoietic enactivism, provides a further way of distinguishing interpersonal affordances from other types in terms of affording possibilities specific to a social interaction. That is, interpersonal affordances are offered in an autonomous interactive process that emerges in the coupling of agents.

The enactivist notion of autonomy is based on the most fundamental of organismic processes: self-maintenance and self-production. These self-organizing processes form the foundation for the autopoietic approach to cognition (Maturana and Varela, 1980). An organism must maintain itself and its boundaries through a network of biological processes while at the same time being selectively open to the world in order to take in from the environment what it needs to sustain its existence.

Summarizing Varela (1979), Thompson (2007, p. 44) describes the autopoietic view as holding that processes constituting the autonomous organization of a system: “(i) recursively depend on each other for their generation and their realization as a network, (ii) constitute the system as a unity in whatever domain they exist, and (iii) determine a domain of possible interactions with the environment.” The autonomous system thus creates the conditions of its own persistence, and the capacities of the system establish the ways in which it can interact with the world.

Maintaining these processes requires that the system be open to the world in ways that enable the system to continue these maintenance processes. Being open to the world in ways that are appropriate for the organism is possible because, in addition to having the capacities to act, organisms are able to make sense of the world in some way. Sense-making (Varela et al., 1991) involves an organism actively exploring a world through the perception of what might be helpful for maintaining organismic integrity and what can hinder or harm, and acting accordingly. Or, more concisely, it is “the creation and appreciation of meaning in interaction with the world” (De Jaegher, 2013b, p. 6).

In the autopoietic tradition of enactivism, an agent can be defined as “an autonomous system capable of adaptively regulating its coupling with the environment according to the norms established by its own viability conditions” (Di Paolo et al., 2017, p. 127). This is not to say that agency itself is attributable to the organism, as enactivism holds that cognition is a relational process rather than involving the internal processing of environmental information. On the enactive account, “perhaps agency is not a property that belongs exclusively to a system but is a property of a *relation* between that system and its surroundings. And this relation is variable” (Di Paolo et al., 2017, p. 110). Thus while we might call an organism an agent, agency itself would be the relational process of selectively attuning one’s actions in accordance with the environment and others. The relational account of agency is variable, in that there is an interactional asymmetry between the organism and the environment, and the relationship fluctuates given the organism’s needs and perhaps environmental demands. There can be a difference in the balance of agency in the agent-environment relationship given the particulars of a current circumstance. For instance, the balance of agency in the agent-environment relationship will be different when I am looking in the fridge for a midnight snack versus when I am fleeing a park due to a sudden high-wind storm.

While these provide a picture of the most minimal processes of life and cognition, these notions scale up to more complex behaviors and systems of organization. For social organisms, the agential process of active attunement does not simply mean that the environment includes others but that others contribute to agential processes and interactions with others can be their own autonomous processes. These interpersonal and social dynamics are captured in the theory of participatory sense-making, as introduced by De Jaegher and Di Paolo (2007). De Jaegher and Di Paolo (2007, p. 497) define participatory sense-making as “the coordination of intentional activity in interaction, whereby individual sense-making processes are affected and new domains of social sense-making can be generated that were not available to each individual on her own.” The interaction is mutually co-constituted, co-regulated, and co-sustained by autonomous agents, who are recursively shaped within the interaction they are sustaining. In participatory sense-making, we have the coupling of autonomous systems that, through that coupling, create an autonomous interaction that involves a precarious balance between participants in order to be maintained.

Being able to be involved in processes of mutual creation of social meaning is important to self-production and maintenance within the intersubjective sphere. It is through these kinds of interactions that the normativity of social practices in the social niche are created, shaped, and changed. For human forms of life, maintaining autonomy involves more than organismic processes of self-production and maintenance in a purely bodily sense. De Jaegher and Di Paolo (2007, p. 493) give a brief description of the criteria for establishing that an interaction is social, based on this interactive notion of emergent autonomy:

“Social interaction is the regulated coupling between at least two autonomous agents, where the regulation is aimed at aspects of the coupling itself so that it constitutes an emergent autonomous organization in the domain of relational dynamics, without destroying in the process the autonomy of the agents involved (though the latter’s scope can be augmented or reduced).”

In participatory sense-making, preservation of the autonomy of the involved agents involves a mutual recognition of the subjecthood of the other. This recognition is meant in an immediate fashion – it is not that one decides the other is a subject, but that they are *already* seen *as* “a subject, not an object” (McGann and De Jaegher, 2009, p. 428; also see Schilbach et al., 2013). To this, I add that this similarly also involves the direct perception of the other as an agent. There is a direct perception of the agency *and* subjectivity of the other.

The interaction process can and does involve asymmetries of autonomy in order to maintain itself. Agency is recognized, while autonomy fluctuates. This is because the interaction process also involves ebbs and flows of mutual regulation (Di Paolo et al., 2018). In an interaction, the regulating role of the processes of mutual sense-making should, ideally, flow back and forth between agents in order to co-constitute the interactive process. This will involve coordination in multiple dimensions. For instance, two people may be engaged in a conversation at a coffee shop. There will be bodily coordination in the sense that they pre-reflectively align their postures (Richardson et al., 2005), and they will perhaps be pre-reflectively balancing their emotional states in response to the other (Hatfield et al., 1993; Kiverstein, 2015). Both participants may pre-reflectively compromise in order to attune to the comportment of the other. One may follow the other in leaning forward when exchanging a particularly juicy bit of gossip or leaning back when talking about how busy their workweek has been. One may have a long story to share, and there may be an asymmetry in regulating the flow of utterances in the interaction – one person is regulating through their continued utterances, while the other is regulated as listener, offering a chuckle or gasp at the appropriate times. While the regulator and regulated roles flow back and forth, neither party’s autonomy is ever harmfully compromised in this idealized example. Both are perceived by the other as autonomous agents within the interaction, both are involved in establishing the norms of that interaction, and regulatory roles can be seen as a matter of request, not force.

Now, consider that affordances are possibilities for action (or interaction). Rietveld et al. (2013, 2017) want to avoid a hard distinction between the perception of social and environmental affordances by appealing to the similarities in *how* we perceive them as embodied agents. Pointing to the Skilled Intentionality Framework, they note that the skill of picking out relevant affordances generates “readiness of the affordance-related ability” (Rietveld, 2008). Whether a relevant affordance is environmental or social, “starting from bodily or skilled intentionality, our perspective avoids an artificial separation between social cognition and nonsocial engagements with the environment” (Rietveld et al., 2013).

This is unproblematic if we are talking about the difference between environmental and social (in the sense of sociability) affordances. However, if we are talking about interpersonal affordances, those afforded *by* or *in interaction with others*, the lack of distinction becomes an issue. First, interpersonal affordances are not given in the relationship between an agent and an environment but in the relationship between agents. De Jaegher and Di Paolo (2007) argue that these are different types of coupling (see also De Jaegher, 2009). The divide between environmental affordances and interpersonal affordances is not artificial – in the first case, you have a mere coupling, and in the latter case, there is a mutually regulated coupling:

“Thus, social interaction has two characteristics: (1) there is a coupling, which is regulated so as to generate and maintain an identity in the relational domain. Thus, the resulting relational dynamics are autonomous in the strict sense of precarious operational closure … and define events and processes as either internal or external to the interaction. And (2) the individuals involved are and remain autonomous as interactors” (De Jaegher and Di Paolo, 2007, p. 493).

The skill of being attuned to relevant affordances should also include a sensitivity to the possibility that one can engage in a social interaction. This would often involve directly perceiving one as an agent able to enter into an autonomous interaction, due to the intertwining of perceiving-as and action-readiness. Perception (on both ecological and enactive accounts) is an active process of looking for action possibilities in the environment, so the perception of interpersonal affordances will often involve specifically looking for affordances provided by an agent. It might be relevant that one is a specific agent (when one has an appointment to meet with a friend), or it might be relevant that one is an adult agent more generally (if I am on the street looking for someone to speak with so I can ask for directions), but nonetheless, I am actively perceiving an agent, and the perception of agency is intertwined with my readiness to respond to a perceived interpersonal affordance.

There are many fairly innocuous reasons that an agent’s autonomy might be compromised in an interaction: we can imagine a caregiver giving a child a stern talking-to for misbehavior, for example.

There are also ways in which sociocultural position, norms, and power dynamics can limit the speech affordances available in some interactions or what a speaker affords to others with their words (Ayala, 2016). A member of a marginalized group, for example, may perceive opportunities to interact differently (or perceive less of them), might find that their words have less impact or that they solicit less attention in interaction with a member of a dominant social group. This could be considered a compromise of autonomy and/or contributor to regulation role imbalance, in that it narrows the possibilities for engaging in collaborative sense-making. There also exist more extreme imbalances in autonomy, in the case that one is *not* treated as a subject and as an agent. These can constitute a grievous devaluation or dehumanization, such as occurs in torture or warfare, where one is treated as non-human (animalistic dehumanization) or as not possessing agency at all (mechanistic dehumanization; Haslam, 2006; Gallagher and Varga, 2014).

Failures to recognize a person as an agent are not only something that happens in these extreme cases though. This frequently happens more subtly in everyday interactions, when failing to recognize one’s agency by perceiving them as an object or tool. Further, there are other harms of recognition, such as failing to recognize another’s social selfhood in interaction. In the final section, I will expand on the ways that neglecting or refusing to perceive one as a self can be an ethical issue. First, though, in the following section, I will describe Maiese’s enactive notion of selfhood (Maiese, 2019) so that we can also look at the importance of perceiving a persisting self in human interactions.

## Enactive and Embodied Selfhood

For human forms of life, agency alone is often not going to be a robust enough notion to capture what it is we might want recognized in social interactions. We are also selves, persisting over time, with particular lived experiences, identities, and ways of being in the world. How it is that we can say a “self” exists, is individuated, and persists over time though is a matter of much contention. The enactive account provides a multi-dimensional and nuanced approach to agency – there are several domains of agency that enable and constrain each other through their overlap of processes and sensorimotor schemes, such as organismic agency (discussed in section “Enactive Autonomy and Interaction”), sensorimotor agency (Di Paolo et al., 2017), and linguistic agency (Cuffari et al., 2015; Di Paolo et al., 2018). The complexity of these latter kinds of agency, their intersubjective development, and their ubiquity in our social niche enables the formation of what Kyselo (2014) has called the socially individuated self. Building on the strengths of Kyselo’s work, Maiese (2019) has proposed a life-shaping thesis of selfhood, grounded in autopoietic enactivism, which like enactive accounts of agency is nuanced and multi-dimensional. I will use the life-shaping thesis here for demonstrating the importance of selfhood in human interactions, in perceiving interpersonal affordances and understanding the ethical aspects of recognizing each other as more than mere agents.

Kyselo’s main concern is that we need a unifying theory of self “as a whole, something that can count as a distinguishable unit of explanation and eventually help to interrelate different aspects of the self” (Kyselo, 2014, p. 2), and that can be used to guide work in the cognitive sciences. She argues that the social self is “never fully separable from the social environment, but instead determined precisely in terms of the types of social interactions and relations of which it is, at the same time, a part” (Kyselo, 2014, p. 12). Kyselo’s answer to the problem of unification distinguishes between two possible answers cognitive scientists might give in trying to locate the self. The first is the idea that what individuates the self is the living body, which she says entails that the social is non-constitutive of the self. The second is to individuate the self as a coherent unity according to the social dimension. She argues in favor of this second option, holding that the social is constitutive of the self.

To think of how the social self is determined in social interactions, we can consider the recursivity in participatory sense-making, where the autonomous agents both shape and are shaped by their social interaction. McGann and De Jaegher (2009, p. 433) say of this process that “[c]ulture transforms our body from a physical mode of cognition, action, and perception to a social one where action can be shared, values coordinated. It is a dramatic alchemy that occurs through participatory sense-making and the acknowledgement of the agency of another. The implications of this fact for the enactive approach cannot be overstressed.” Thinking about social selves and the ethical dimensions of interactions is one way of taking up these implications.

However, Maiese (2019) points out that the theory of participatory sense-making only goes so far as to say that social interactions shape the participants, not determine them. Instead, Maiese offers a “life-shaping thesis” of selfhood, in which the self is individuated by the body while being shaped by the social, to account for the unification of the self over time. While Kyselo (2014) claims that the self is individuated *via* social relations, rather than *via* the body, Maiese (2019, p. 364) bases selfhood in the autonomous organization of a system, which requires that an organism individuates itself as a closed network of systems of self-maintenance. She holds that the individuated self “is fully embodied, and that the various dimensions of mindedness—that is to say, our desires, feelings, emotions, sense perceptions, memories, thoughts, intentional actions, etc.—are all partially determined, or shaped, by the social world” (also see Scheman, 1983). For humans, the intersubjective scale of agency involves individuating oneself in the social realm, but this is scaffolded by the ongoing bodily processes by which we are able to maintain our individuation over time. So while the self is shaped by the social, this does not root the persistence conditions of the self in the social. Rather, the social would be one domain of embodiment of the organismically individuated self, which would enable and constrain other dimensions of embodiment.

Maiese’s proposal of the life-shaping thesis (Maiese, 2019) provides a robust enactivist notion of the self that does not make us choose between the self of cognitive science and the social self. The enactive account holds that cognition is constituted by a number of nested processes, involving body, brain, and world – and for humans and other social organisms, shaped intersubjectively. Though Kyselo frames the discussion of the self in terms of a context/constitution dichotomy, the project of deciding between the social as contextual or constitutive of the self is perhaps a bit misguided in terms of metaphysical presuppositions. Enactivism, as a non-reductive, process-oriented, intersubjective, and multiscale account, can accommodate the social as both contextual and constitutive of the self in various ways, as Maiese shows. Further, individuation – differentiating between self and environment – has been held as one of the main characteristics of agency for minimal autopoietic systems (Di Paolo, 2005; Barandiaran et al., 2009). It is not quite clear why another sense of individuation would be necessary. On this, Maiese (2019, p. 364, emphasis added) says:

“This distinction between components that constitute the living system and elements that form its environment grounds not only biological identity, but also the identity of the self. Indeed, just as a living system should be individuated according to this form or organization, the self (or what might described as the human *mode of life*) should be individuated according to its characteristic form or organization, rather than the energetic or relational material that ensures its continued existence.”

Maiese seems to ground unification in both the individuating (physical) and persisting (temporal) sense in the autonomous processes of living systems, in line with the autopoietic notions of individuation through self-maintenance and self-production. The life-shaping thesis holds that the social is not constitutive of the self, but that the self is fundamentally *embedded* in the social. The self, she argues, is influenced and shaped by the social in the sense that the social has a causal influence, is reciprocally shaped by us through our responses or contributions to the social, and is normative. It is normative because the social shapes our internal norms not only through enabling or constraining our embodied processes but also in the contributory sense of taking part in participatory sense-making and practices that can reinforce, shape, or transform social norms. In this way, through social participation and self-shaping, social normativity is recursive.

Grounding the self in this way is important for my account for two reasons in particular. First, understanding the self as fundamentally embodied does not allow for full determination of what unifies the self over time in the social. To say this is perhaps too dismissive of the first-person authority we have on our own existential identities (Bettcher, 2009) and the way these identities shape how we extend ourselves (through our aims, plans, and goals) into the future (Brancazio and Segundo-Ortin, 2020). Relatedly, it is only because we *can* act out of accordance with social expectations and demands that we have the means for transformative change *of* the social. Second, because it preserves agency and autonomy, the life-shaping thesis can be productively integrated with the enactive theories of participatory sense-making and direct perception in interaction. The self is engaged in social interactions in which it can be shaped or influenced, but it is not fully determined within the sphere of these acts, thus fundamentally preserving the autonomy of the embodied agent. Maiese (2019, p. 363) voices similar concerns about Kyselo’s determination of the self in the social and the implications for compatibility with participatory sense-making:

“…indeed, participatory sense-making presupposes and requires bodily-organismic ‘selves’ who can partake in the interaction process. Moreover, for each of these ‘selves’ to remain an autonomous interactor, it must be possible (even if unlikely) for her to defy social expectations, or even disengage from the social interaction if she feels so inclined.”

It is also important to note that by being accommodating to varying socioculturally situated notions of self, this does not necessarily mean that individuals have *a* self in the narrative or reflective sense. In other words, I believe we can take Maiese’s notion of selfhood as not implying that the social self is necessarily unified, or unified in any particular way, *apart* from the embodied sense^2^. A persistent theme in feminist theory and critical race theory is multiplicitous selves and identities. Given the numerous communities that one may navigate in their social terrain, one may have the experience of enacting, adopting, and being treated as more than one social self – especially in the case that one belongs to one or multiple marginalized groups (e.g., Anzaldúa, 1987; Harris, 1990; Wing, 1990; Ortega, 2001; Barvosa, 2008). In fact, in this work, it is oftentimes embodied persistence through multiple social worlds, or the phenomenological *mine-ness* of experience given through embodied persistence and subjectivity, that is said to ground individuation or persistence conditions through which the agent is able to enact numerous selves in the social sphere (Alcoff, 2006). Locating the individuation and persistence of selfhood in the “self-organizing” of autonomous systems opens up room for an enactive approach to how it is that selves can manifest in different ways, depending on particularities of context, social roles and cultural knowledge, power dynamics, marginalization and oppression, and other aspects that shape the way that an agent will take up an interaction.

The notion of selfhood proposed by Maiese (2019) captures the root of what is important for developing an account of how it is that we directly perceive and selectively respond to interpersonal affordances. On her account, the social self is an aspect of the embedded embodied self, and the persistence conditions of selfhood, while socially embedded, are maintained by the embodied processes of organization rather than being fully socially determined. The subject directly perceived in participatory sense-making is an embodied subject embedded in the social. Further, the account makes no general claims about what social selfhood is and can be sensitive to the myriad ways that sociocultural norms, practices, multiplicity, and neurodiversity can influence self-perception and experience.

## Interpersonal Affordances Between Agents and Selves

I will turn back now to the direct perception of agency and selfhood in the social sphere by way of interpersonal affordances. As discussed in section “Social Affordances and Interpersonal Affordances,” we should take interpersonal affordances to mean *actual* possibilities for interaction *with an agent*. An interpersonal affordance is not perceived in the agent-environment relationship, but is afforded *by* another agent (whether intentionally or not). Interpersonal affordances are not necessarily already part of an interaction, but they can afford an interaction. For example, let us say that I am walking down the street and I see a friend, who is engaged in a conversation with someone else. I may perceive them as affording a social interaction, though they have not actually seen me yet – so there is no intention on their part *to* interact. Conversely, in participatory sense-making, both agents are actively affording possibilities for interaction through their ongoing utterances, gestures, bodily and emotional coordination, and so on. In both cases, the perception of interpersonal affordances is not a product of the agent-*environment* relationship, but of the agent-*agent* relationship, and involves seeing the other as a subject.

In section “Social Affordances and Interpersonal Affordances,” I explained that interpersonal affordances are directly perceived: “The sight of a sad friend affords consoling him or her, a colleague at the coffee machine solicits small talk, and an extended hand immediately prepares the body for shaking it” (Rietveld et al., 2013, p. 436). It is crucial to note that in this example, the *perception-as* and the *action-readiness* are intertwined, as with the direct perception in the interaction theory of social cognition (see De Jaegher et al., 2010; Gallaghehr and Varga, 2014). However, the perceiving-as in interaction is not perception of a static state. Fiebich (2014, p. 1) makes the point that interpersonal affordances are “perceived within interactive reciprocal processes,” where the perceived agent is engaged in ongoing action processes in response to the behaviors of the other in interaction. This is also argued for by McGann (2014, p. 26): “There is also no particular moment in time at which perceiving is ‘complete’ because such perception always occurs in the flow of on-going behavior – activity does not have to wait for it.” A continuous interaction offers a continuous stream of changing interpersonal affordances – and, recursively, engagement with these affordances changes the process of interaction.

The participatory sense-making account provided in section “Enactive Autonomy and Interaction” makes it clear that these reciprocal processes often happen within an autonomous interaction, where the interactors are involved in a shared, co-regulated (and co-regulating) domain of sense-making. Taking this into account, perceiving what is afforded by the other agent can also be influenced by the perceiver’s desire to maintain the interactive coupling. The perception of relevant interpersonal affordances by each individual agent will involve more than the concerns of their own self-maintenance – they include concerns about the maintenance of the autonomous interaction as well. Or, perhaps, the relevance of affordances will instead be influenced by an agent’s desire to leave the interaction (so they may begin glancing around the room, looking at their phone, or become slow to respond to the interpersonal affordances the other agent is offering).

As discussed above, participatory sense-making requires seeing another as a subject. In other words, maintenance of an autonomous interaction, or agent-agent coupling, already presumes agency.

My claim here has been that interpersonal affordances in participatory sense-making also involve the direct perception of agency and, to some extent, selfhood. This, I believe, has some ethical implications, in line with the ethical dimensions of the Gibsonian perspective of affordance perception: “The meaning or value of a thing consists of what it affords. What a thing is and what it means are not separate, the former being physical and the latter mental as we are accustomed to believe” (J. Gibson, 1982, p. 407). If we apply this to interpersonal affordances, we can consider how being seen as an autonomous agent capable of entering into a participatory sense-making process would be a valuation of our contributions to that shared domain of sense-making. Thus, while it does not matter to an apple whether it affords edibility to a person, it can matter immensely whether a person is viewed as a candidate for shared meaning-creation or shaping. The discussion of interpersonal affordances thus must involve examining how prejudices, power dynamics, biases, and social status influence how one is perceived and how this affects their ability to contribute to participatory sense-making.

On the farther end of compromises of agency in interaction, we can think of objectification. Objectification has many aspects, which have been detailed by Nussbaum (1995) and Langton (2009). The most important of these aspects for understanding the relationship between objectification and interpersonal affordance perception is the denial of autonomy, being treated as a tool or a means to an end and the treatment of someone as interchangeable with objects (or fungibility) (Nussbaum, 1995), as well as reduction to body and/or appearance (Langton, 2009). Black feminism has long brought attention to the objectification and dehumanization that Black women experience, especially in terms of animalistic dehumanization and the denial of agency (Rollins, 1985; Collins, 1986; Crenshaw, 1991). These kinds of experiences (and others, such as objectification through fetishization) have also been discussed in trans theory, most predominantly in the experiences of trans persons of color (Flores et al., 2018).

Let us consider a serious case of objectification: street harassment (which may include misogynistic, racist, ableist, transphobic, classist, or queerphobic harassment, as well as many intersecting combinations of these). This kind of harassment usually involves a stranger uttering derogatory or sexual words of phrases to an individual, though this can also take place through (or include) stares, ogling, or physical menacing. In describing the psychological effects of the sexual street harassment of women, Davis (1994, p. 143) says that it “allows men to establish the boundaries of participation in the street. …Through street harassment, men inform women that women are public participants only with men’s permission.” It is perhaps obvious that the individual being harassed is not perceived by the perpetrator as affording the kind of treatment that appropriately acknowledges their agency and autonomy.

This is neither a social interaction nor an invitation to create a shared domain of meaning. Objectification of this kind, seems to be more akin to a “skill” if we are using the Skilled Intentionality Framework (van Dijk and Rietveld, 2017). The perpetrator views the harassed person as a relevant affordance, not for a social interaction but for objectification (dehumanization, denial of agency, being treated as a means to an end, and so on). That is, given the above discussions about direct perception in ecological and enactive approaches, we can think of the action-readiness tied to the perception of a marginalized agent as an opportunity to enact a skill (explicitly or implicitly) intended to foreclose the possibility of meaningful participation. However, we should be cautious about going too far in explaining objectification through what is exercised by an individual, as this places too much responsibility on the individual perpetrator when we should also be looking at the systemic issues and social structures that allow (or encourage) this type of treatment to become habituated.

It may often be appropriate to objectify the local environment as affording something for you, within reason and given prevailing norms. It is not appropriate to perceive an agent as offering something for you in the same way, if it constitutes a devaluation of the person^3^. But even these are the situated claims of a Western anglo philosopher – the environment/interpersonal distinction, and appropriate attributions of agency and autonomy in perception, may be very different in other cultures (Kelly and Lobo, 2020), in which case we ought to look at how affordance perception in those cultures is socially shaped (as discussed in section “Social Affordances and Interpersonal Affordances”) and be ready and willing to adjust our theories about affordance perception accordingly.

We also need to take into account that, as previously discussed, interactions do not just take place between ahistorical agents. I have argued that participatory sense-making involves the coupling of selves in the interaction process. This means that there is a recognition not just of an embodied agent in the course of interaction, but also a socially embedded agent – an agent that has a way (or ways) of being in the world with others that pre-exists and continues on after the interaction. I hold that persisting embodied selfhood, as discussed by Maiese (2019), is directly perceived rather than reflectively attributed or inferred. While this is not the case for every interaction, I think this is an important aspect of participatory sense-making. Seeing the participant as an embodied, socially embedded self allows for the coordination of expectations about shared meanings that structure the interactive space. And in creating a shared domain of sense-making, there are opportunities for creating and shaping meaning for the social self that extend beyond the interaction itself.

In contrast, one who is denied aspects of their selfhood is subject to a compromise in their autonomy in participatory sense-making. One way this might manifest is through the denial of interpersonal affordances to those belonging to non-dominant or oppressed groups. Of course, while street harassment is an obvious harm, there are more systemic and pervasive ways in which non-dominant groups are not perceived as full agents or selves. Speaking on the narrator of Ellison’s *Invisible Man*, Charles Mills discusses the experience of this kind of ongoing racialized objectification:

“His problem is his ‘invisibility,’ the fact that whites do not see him, take no notice of him, not because of physiological deficiency but because of the psychological ‘construction of their *inner* eyes,’ which conceptually erases his existence. … So his problem is to convince them that he exists, not as a physical object, a lower life form, a thing to be instrumentally treated, but as a person in the same sense that they are, and not as a means to their ends” (Mills, 1998, p. 9; quotes from Ellison, 1952).

Another example of a more deliberate denial of selfhood would be engaging in an interaction with a person but consistently not using their pronouns. To do so is to perceive one as a social self, with an autonomous identity, and then purposefully undermine that very sense of self in the process of interaction through the interpersonal speech affordances offered. Insisting on denying someone’s selfhood in interaction in this and other ways denies full entry into participatory sense-making, as it is a forced regulation of autonomy. This kind of harm, as a denial of selfhood and agential identity (Barnes, 2019; Dembroff and Saint-Croix, 2019), limits an agent’s ability to participate in the co-creation of meaning (De Jaegher et al., 2016) in a social interaction, among causing or perpetuating other harms.

In closing this section, I believe it is important to note that looking at the experiences of those who have their agency and selfhood actively denied suggests that we ought to be very careful about what we can take for granted in enactive and ecological approaches to social interactions and affordance perception. While there is clearly more to discuss in regards to the perception and denial of agency and selfhood, my intention has been to demonstrate that the direct perception of interpersonal affordances involves the perception of agency. I have also argued that in many forms of interaction, including participatory sense-making, direct perception will also involve seeing the other as a self. To not appropriately perceive these in some cases can constitute serious harms to a person. Using the enactive theory of participatory sense-making, I have also shown that this can limit one’s ability to enter into processes of meaning creation or shaping.

## Conclusion

As enactivism and ecological-enactivism progress in explaining complex human realms of being, they grow increasingly concerned with social normativity and social institutions. For example, De Jaegher (2013b) has looked at how patriarchal and democratic institutions can be understood through the enactive approach to intersubjectivity. Maiese and Hanna (2019) have offered concrete suggestions for transforming our political and social institutions using insights from enactivism and ecological psychology. And Rietveld et al. (2017) have brought attention to the important challenge of adapting insights from enactive and embodied cognition into resources for increasing social cohesion and inclusivity.

I have argued that recognition of selfhood and maintaining a distinction between environmental affordances and interpersonal affordances are important for these projects. On one hand, this is explanatorily important due to the different kinds of coupling involved. On the other hand, this distinction is important for theorizing about the ethical and political aspects of affordances. To say that perception of affordances is the same, whether environmental or social, generalizes away from the concrete realities of experience and selfhood in interaction.

If we are looking for ways to increase social cohesion “understood as the co-existence of disparities, not the elimination of particular backgrounds” (Rietveld et al., 2017, p. 303), as Rietveld et al. have discussed, we first need to understand the concrete particularities of bringing people together in social spaces. In bringing together ecological and enactive approaches to evaluating the ways in which our social institutions and practices can be transformed, we must also actively build resources for examining and understanding how our habits and actions contribute to devaluation and other harms to other agents. And by being attentive to the ways in which marginalization and oppression structure social interactions, we can better examine the ethical aspects of our research on interactions, as well as practicing more ethical interactions ourselves.

## Author Contributions

The author confirms being the sole contributor of this work and has approved it for publication.

### Conflict of Interest

The author declares that the research was conducted in the absence of any commercial or financial relationships that could be construed as a potential conflict of interest.

## Footnotes

^1^Eleanor Gibson is often overlooked and under-cited, and oftentimes a citation to “Gibson” is assumed to be a reference to another well-known Gibson. Following the convention introduced by Miguel Segundo-Ortin, I cite Eleanor Gibson as “E. Gibson” and James Gibson as “J. Gibson” to bring more attention to her unique contributions.

^2^There is quite a bit of literature on narrative selfhood and the distinction between minimal and narrative selves, which I will not be taking up here. Maiese’s account provides a way of having a unified embodied self without necessarily making commitments to any specific (or even unified) reflective or narrative self (for more discussion on this distinction, see Menary, 2008; Mackenzie, 2014, or Zahavi 2007).

^3^This would not apply in cases such as stopping someone on the street to ask the time, as this is a request for assistance, not a denial of agency.
